# Effect of vitamin C and hesperidin on serum uric acid concentrations in healthy adults with high uric acid levels: the randomized controlled ‘HesperidrinC trial’

**DOI:** 10.1007/s00394-026-03905-z

**Published:** 2026-02-25

**Authors:** Janna Enderle, Rebecca Dörner, Daria Tondar, Mario Hasler, Caroline Gilcher, Christof B. Steingass, Ralf Schweiggert, Manfred J. Müller, Anja Bosy-Westphal

**Affiliations:** 1 Institute of Human Nutrition and Food Science, Christian-Albrechts University Kiel, Düsternbrooker Weg 17, 24105 Kiel, Germany; 2 Applied Statistic, Agricultural and Food Economics Faculty, Christian-Albrechts University Kiel, Kiel, Germany; 3 Chair of Analysis and Technology of Plant-based Foods, Department of Beverage Research, Geisenheim University, Geisenheim, Germany

**Keywords:** Serum uric acid, Uric acid excretion, Hyperuricemia, Vitamin c, Hesperidin

## Abstract

**Purpose:**

While orange juice has been reported to decrease serum uric acid (SUA), the effects of the specific constituents hesperidin and vitamin C are not fully understood. The present study aimed to unravel their possible SUA-lowering effects.

**Methods:**

In a randomized controlled, double-blind, two-way cross-over, two-week intervention, the effects of four orange-flavored drinks (200 mL/d) on SUA (primary outcome) were compared in 40 adults (21–78 y; BMI 17.9–41.4 kg/m^2^) with high SUA. One drink was a placebo; the others contained 600 mg vitamin C (*VitC*), 240 mg hesperidin (*Hesp*), or both (*VitC+Hesp*). Blood and urine concentrations of uric acid, vitamin C, and hesperidin metabolites were measured, along with the HOMA index as a potential confounder.

**Results:**

*VitC* increased plasma vitamin C levels, *Hesp* increased urinary excretion of hesperidin metabolites, and *VitC+Hesp* increased both. Higher baseline plasma vitamin C levels resulted in diminished increases in vitamin C (*VitC*: *r* = -0.53; *p* = 0.02 and *VitC+Hesp*: *r* = -0.79; *p* < 0.001). Levels of SUA decreased in response to *VitC* and *VitC+Hesp* with no changes in urinary uric acid excretion (UUA) or clearance (UAC). The increase in plasma vitamin C was associated with a decrease in SUA (*r* = -0.25; *p* < 0.03) with larger effects at higher baseline SUA (*r* = -0.24; *p* = 0.03). A high HOMA index was associated with lower plasma vitamin C and elevated SUA.

**Conclusion:**

Vitamin C but not hesperidin decreased serum uric acid levels without affecting uric acid excretion.

**Clinical Trials registration:**

www.clinicaltrials.gov; NCT04316390; July 15, 2022.

**Supplementary Information:**

The online version contains supplementary material available at 10.1007/s00394-026-03905-z.

## Introduction

Variation in serum uric acid (SUA) may result from its increased endogenous production, impaired renal excretion, or a combination of both. Hyperuricemia, defined as uric acid concentration approaching the limit of urate solubility (i.e. approximately 7 mg/dL; 0.416 mmol/L), is a well-documented risk factor for the development of gout. However, there is a growing body of evidence that suggests a link between high SUA levels and cardiovascular disease and type 2 diabetes [1–7]. In Germany and the US, approximately 20% of adults have elevated uric acid levels, while around 1–2% of German adults and 3.9% of US adults suffer from gout [8, 9].

As a dietary approach, a low purine diet (from animal sources) and the avoidance of alcoholic beverages and soft drinks have been recommended for the management of hyperuricemia [10]. In contrast to sugar-sweetened beverages, two weeks of controlled consumption of 1.2 ± 0.2 L bottled orange juice per day was associated with a decrease in SUA [11]. Similarly, Morand et al. (2011) have reported a decrease in SUA following the consumption of 500 mL of an orange juice [12]. The observed effects can be explained by the uricosuric effect of vitamin C [13–16], and/or by a flavonoid-induced competitive inhibition of xanthine oxidase, which consequently reduces the endogenous production of uric acid [17, 18]. However, the effects of specific constituents of orange juice (i.e. hesperidin, vitamin C, and a combination of the two) on lowering SUA have not yet been investigated. The present study, therefore, aimed at unraveling the possible SUA-lowering effects of both orange juice constituents. To this end, a randomized controlled, double-blind, two-way cross-over, two-week intervention was conducted, during which four juice-like beverages were compared. One drink served as a placebo and, therefore, contained neither hesperidin nor vitamin C (*Control*). The other drinks contained either hesperidin (*Hesp*) or vitamin C (*VitC*) or both (*VitC+Hesp*). Throughout each intervention period, the levels of uric acid, vitamin C, and hesperidin metabolites in blood and urine, respectively, were measured. In addition, insulin resistance (IR) was assessed as a possible confounder.

## Methods

**Fig. 1:**
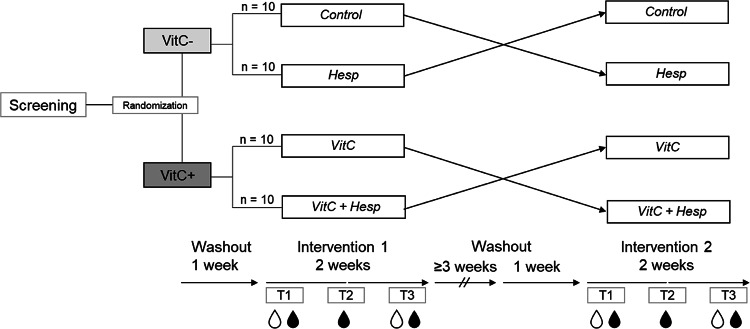
Design of the randomized controlled, double-blind, two-way cross-over, two-week intervention ‘HesperidrinC study’. blood sampling; 24 h urine collection

The ‘HesperidrinC trial’ was a randomized controlled, double-blind, two-way cross-over, two-week intervention study (Fig. 1) conducted between August 2022 and March 2023 at the Institute of Human Nutrition at Kiel University.

A total of 40 study participants were recruited from the database of a previous study as well as from students and staff of the University of Kiel via email. Public information was distributed at the University of Kiel, the University of Applied Sciences, and the University Hospital Campus Kiel. Women and men with SUA levels > 4.5 and > 5.5 mg/dL, respectively, were identified as eligible to participate. Exclusion criteria were: age under 18 or older than 80 years, diagnosis or history of gout attacks, medical treatment for high blood uric acid level, active cancer treatment, diagnosis of diabetes, fructose intolerance, anemia or iron deficiency, smoking, current pregnancy or breastfeeding, weight loss of > 5% during the past three months, ascorbic acid supplementation, concurrent participation in another intervention study. People interested in participation were invited to the Institute of Human Nutrition for full study information and uric acid blood screening.

All participants provided fully informed and written consent before inclusion. The study was approved by the ethics committee of the Christian-Albrechts-University Kiel (AZ: D 402/20, 22.08.2022) and has been performed in accordance with the Declaration of Helsinki [19].

**Fig. 2:**
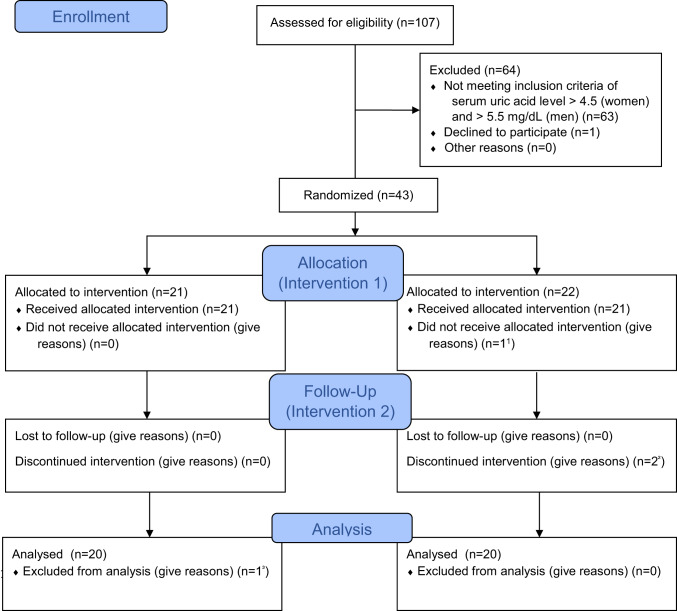
CONSORT flow-chart of included and excluded participants over the course of the study.^1^One participant declined participation beforethe first treatment. ^2^One participant developed an irritant skin reaction. ^3^Incidental finding:Diabetes Type 2- not meeting inclusion criteria

Of those screened for eligibility, 63 participants were not included in the trial due to SUA levels < 4.5 and < 5.5 mg/dL in women and men. Participants who met the inclusion criteria were randomly assigned to one of the two study arms, stratified by age, sex, and screening SUA level. Each study arm comprised two groups, i.e., study drink intervention periods of two weeks each, conducted in random order and double-blind cross-over design (for CONSORT flow chart see Fig. 2).

One participant discontinued after one week due to an irritant skin reaction of unknown origin.

### Study procedure

Participants underwent two interventions separated by at least four weeks, with a one-week washout period before each intervention. They refrained from consuming alcohol, citrus fruits and citrus products, and vitamin C-enriched foods and supplements during washout and intervention periods. The study procedure is shown in Fig. 1. Participants came to the institute after an overnight fast for blood sampling at the start of the intervention (baseline, T1). They were provided with their randomly assigned study drinks for the next six days and were instructed to consume one drink of 200 mL each morning with breakfast after shaking the bottle vigorously. After one week, they returned to the institute for another fasting blood sampling (T2) and provision of further study drinks for the remaining intervention period. The final fasting blood sample was taken after week 2 (T3). Participants collected their urine during 24 h prior to the assessments at T1 and T3. Body mass index (BMI, kg/m^2^), body composition (as assessed by Bioelectrical impedance analysis, mBCA 515, seca, Hamburg, Germany [20]), blood pressure, and vascular stiffness (Vicorder^®^, SMT Medical, Würzburg, Germany) were assessed.

### Study drinks

Study drinks were designed and produced at Geisenheim University and Eckes-Granini Group (Nieder-Olm, Germany), respectively. All drinks had essentially the same base recipe and were respectively enriched with vitamin C, hesperidin, both or none of the two to finally yield four comparable, blinded study drinks (see supplemental Table S1). One drink containing neither vitamin C nor hesperidin served as *Control*. Levels of active ingredients, i.e. hesperidin and vitamin C, determined by HPLC-DAD (International Federation of Fruit Juice Producers, 2017 [21]) and iodometry [22], respectively, were monitored at 6 time points over the entire study duration of 56 weeks. The serving of 200 mL of the hesperidin-rich drink (*Hesp*) contained 242 ± 6 mg, equaling 397 ± 10 µmol hesperidin (Hesperidina 2 S, Ferrer HealthTech, Beniel, Spain). The vitamin C-rich drink (*VitC*) fortified with L-ascorbic acid (Döhler, Darmstadt, Germany) on average provided 592 ± 12 mg vitamin C. *VitC+Hesp* contained 241 ± 7 (396 ± 12 µmol) and 591 ± 13 mg hesperidin and vitamin C per 200 mL, respectively. Dosages of hesperidin and L-ascorbic acid approximated those of approx. 1.2 L orange juice, exerting SUA lowering effects in a previous study [11]. When compared amongst each other and to an equal amount of a common orange juice from concentrate, all drinks had an identical calorie content. Glucose, fructose (both Döhler, Darmstadt, Germany), and sucrose (Pfeifer and Langen, Cologne, Germany) were added at 22, 22, and 48 g/L (total sugar of approx. 92 g/L), resulting in a daily amount of sugar of approx. 18.4 g. Citric acid was implemented at 7.0 g/L (Laiwu Taihe Biochemistry, Ji’nan, China). Food colors (safflower extract, 400.5 mg/L and sunset yellow (E110), 2 mg/L), a clouding agent (orange terpene based, 0.6–4.6 g/L in the drinks with or without hesperidin, respectively), and natural orange aroma (0.150 mL/L) (all from Döhler, Darmstadt, Germany), were used to standardize the visual appearance and flavor of all beverages. All drinks were pasteurized and hot-filled into 200-mL clear glass bottles. Study drinks were delivered blinded regarding their composition to the Institute of Human Nutrition at Kiel University and stored in a refrigerator upon provision to participants. Participants and study staff were unable to distinguish between the four test drinks.

### Blood and urine sampling

Following comprehensive study information, blood samples were taken for assessment of SUA level and hemoglobin (Hb)-level to screen for eligibility to participate in the study. Fasting blood samples were taken at the start of each intervention period, after one week, and after two weeks. Urine was collected for 24 h at the start and end of each intervention (i.e., T1, T3, see Fig. 1). SUA and urine uric acid (UUA) concentrations were measured chromatically (BLOSR6 × 98 08, Beckman Coulter, Inc., U.S.A.). Uric acid clearance (UAC) was calculated using the following formula [23]:

$$\:\mathrm{UAC\:[mL/min]\:=\:}\frac{\mathrm{UUA\:[mg/100\:mL]\:*\:urine\:volume\:[mL/min]}}{\mathrm{SUA\:[mg/100\:mL]\:}}$$

In order to assess IR, the Homeostasis Model Assessment (HOMA) index was calculated from fasting glucose and insulin concentrations [24].

### Sample preparation and analysis of urinary hesperidin metabolites and plasma vitamin C

For vitamin C analyses, EDTA-plasma obtained by centrifugation (4 °C, 10 min at 2000$\:\times\:g$) was combined with an equal volume of an aqueous 10% w/v *meta*-phosphoric acid containing 2 mmol/L EDTA, followed by centrifugation (4 °C, 10 min at 16,000$\:\times\:$g). Aliquots from the supernatant were drawn and frozen immediately with liquid nitrogen. Vitamin C in plasma was quantitated by HPLC-fluorescence detection according to Tessier et al. (1996) [25].

For the determination of hesperidin metabolites, 24 h urine was collected and stabilized with 1.0 g of L-ascorbic acid (purity > 99%; Roth, Karlsruhe, Germany). Aliquots were stored at -40 °C and were membrane-filtered using 0.45 μm regenerated cellulose prior to analyses. Hesperidin metabolites in urine were identified and quantitated using an Elute SP (Bruker Daltonik, Bremen, Germany) with ultra-high-performance liquid chromatography system coupled to diode array detection and a *tims*TOF Pro 2 (Bruker Daltonik, Bremen, Germany) high-resolution tandem mass spectrometer with electrospray ionisation (UHPLC-DAD-ESI-QTOF-HR-MS/MS). Chromatographic separation was achieved using a C18 column (Luna^®^ Omega, 150 × 2.1 mm i.d., particle size 1.6 μm, Phenomenex, Aschaffenburg, Germany) and a gradient elution based on MS grade water and acetonitrile, both acidified with 1% of formic acid, at a flow rate of 0.4 mL/min as eluents. MS settings were as follows: Scan range, *m/z* 20–1300; collision energies, 20–50 eV (stepping); nebulizing gas (N_2_), 2.8 bar; dry gas flow rate (N_2_), 7.0 L/min; nebulizer temperature, 225 °C; capillary potential, 3600 V. TIMS settings: TIMS gas, N_2_; scan range 0.10–1.50 V×s/cm^2^.

For the present study, total levels of two specific hesperidin catabolites, i.e., 3-(3ʹ-hydroxy-4ʹ-methoxyphenyl)hydracrylic acid ([M–H]^−^ at *m/*z 211.0614, C_10_H_11_O_5_^–^, indicative MS/MS fragment ions: *m/z* 149.1 ([M–H−CO_2_−H_2_O]^−^)) and 3-(3ʹ-hydroxy-4ʹ-methoxyphenyl)propionic acid ([M–H]^−^ *m/*z 195.0664, C_10_H_11_O_4_^–^, MS/MS: *m/z* 136.1 ([M–H−CO_2_−CH_3_]^•−^)) were considered. In addition, six conjugates, i.e., two isomeric hesperetin-*O*-diglucuronides ([M–H]^−^ at *m/z* 653.1358 and 653.1359, C_28_H_29_O_18_^–^, MS/MS: *m/*z 477.1 ([M–H−glucuronyl]^−^), 301.1 ([M–H−glucuronyl−glucuronyl]^−^)), hesperetin-*O*-glucuronide-*O*-sulfate ([M–H]^−^ at *m/z* 557.0609, C_22_H_21_O_15_S^–^, MS/MS: *m/*z 477.1 ([M–H−SO_3_]^−^), 381.0 ([M–H−glucuronyl]^−^), 301.1 ([M–H−glucuronyl−SO_3_]^−^)), hesperetin-7-*O*-glucuronide ([M–H]^−^ at *m/z* 477.1038, C_22_H_21_O_12_^–^, MS/MS: *m/*z 301.1 ([M–H−glucuronyl]^−^)), hesperetin-3ʹ-*O*-glucuronide ([M–H]^−^ at *m/z* 477.1041, C_22_H_21_O_12_^–^, MS/MS: *m/*z 301.1 ([M–H−glucuronyl]^−^)), and hesperetin-3ʹ-*O*-sulfate ([M–H]^−^ at *m/z* 381.0268, C_16_H_13_O_9_S^–^, MS/MS: *m/*z 301.1 ([M–H−SO_3_]^−^)) were determined. The targeted analytes have been previously reported as characteristic hesperidin metabolites [26, 27]. Quantitation was based on linear calibrations with authentic reference standards using extracted ion current chromatograms of the deprotonated molecules ([M–H]^−^). Hesperetin-*O*-diglucuronides and hesperetin-*O*-glucuronide-*O*-sulfate were quantitated using hesperetin-3ʹ-*O*-glucuronide. Hesperetin-3ʹ-*O*-sulfate was quantitated using hesperetin-7-*O*-sulfate.

### Statistics

The primary outcome of the study was SUA, and the secondary outcome variables were UUA and UAC. The required sample size was calculated to detect a decrease in SUA with an effect size of 0.77 according to our previous study [11] using G*Power 3.1.9.7 software (written by F. Faul, University of Kiel, Kiel, Germany). A significance level of 0.05 and a power of 95% were assumed. Data analysis was performed with the SPSS software package (IBM Corp. Released 2020. IBM SPSS Statistics for Windows, Armonk, NY: IBM Corp; version 28.0) and R (version 4.3.2). The participants were analyzed as randomized. For the characteristics of the study population, the normal distribution was checked via the Shapiro-Wilk test. These characteristics are given as mean ±standard deviation (SD) and range with differences between men and women being assessed with paired t-test or Mann-Whitney-U-test. Associations between levels of and changes in levels of vitamin C, hesperidin metabolites, and uric acid in blood and urine, respectively, and HOMA index were tested using Pearson’s or Spearman’s correlation. A p-value of < 0.05 was considered to be statistically significant.

For the primary outcome variable (i.e., SUA) and secondary outcome variables (i.e., UUA and UAC) as well as for variables displaying intervention effect (i.e., vitamin C in plasma, hesperidin metabolites in urine), the data evaluation started with the definition of a first appropriate statistical mixed model [28–30]. This model included the fixed factors vitamin C, hesperidin, time point, and sex, as well as all their interaction terms, intervention period, and the covariates age and HOMA index. The participant was regarded as a random factor. Also, the (auto-) correlations of the measurement values due to the several time points were considered. The residuals were assumed to be normally distributed and to be homo- or heteroscedastic (if necessary). These assumptions are based on a graphical residual analysis. Based on this model, multiple contrast tests were conducted [31, 32].

## Results

### Baseline characterization

Baseline characteristics of 23 women and 17 men who completed the ‘HesperidrinC study’ are shown in Table 1. Mean age was 50 years and BMI was 27.3 kg/m^2^ with no difference between women and men. One participant was underweight (BMI < 18.5 kg/m²), the prevalence of normal weight, overweight, and obesity was 37.5%, 32.5%, and 27.5% respectively. At T1, SUA concentrations were lower in women than in men, while plasma vitamin C levels were significantly higher in women than in men (38.21 ± 8.77 vs. 32.86 ± 6.89 µmol/L; *p* = 0.004). 18 participants had a HOMA index greater than 2.5, and one participant was diagnosed with type 2 diabetes shortly after entering the study.

**Table 1:** Baseline characterization of the study population of the ‘HesperidrinC study’

	Women	Men	Total study population
	*n* = 23 (57.5%)	*n* = 17 (42.5%)	*n* = 40
	mean ±SD (range)	mean ±SD (range)	mean ±SD (range)
Age, years	48.7 ±19.8	51.9 ±21.0	50.1 ±20.1
	(21–78)	(23–75)	(21–78)
Weight, kg	**77.5 ±17.6**	89.4 ±16.0*	82.6 ±17.7
	**(47.2–112.9)**	**(62.9–133.1)**	(47.2–133.1)
BMI, kg/m²	27.6 ±6.4	27.0 ±4.5	27.3 ±5.6
	(17.9–41.4)	(19.7–36.7)	(17.9–41.4)
FMI, kg/m²	**11.1 ±5.2**	7.5 ±3.2*	9.6 ±4.8
	**(2.8–23.4)**	**(2.6–13.7)**	(2.6–23.4)
FFMI, kg/m²	**16.5 ±1.7**	19.5 ±1.9***	17.8 ±2.3
	**(13.9–20.1)**	**(16.3–23.0)**	(13.9–23.0)
Insulin, µU/mL	10.8 ±7.9	11.1 ±6.2	10.9 ±7.1
	(3.8–37.6)	(4.4–29.3)	(3.8–37.6)
Glucose, mg/dL	97.4 ±8.5	102.3 ±11.6	99.5 ±10.1
	(84.0–120.0)	(80.0–129.0)	(80.0–129.0)
HOMA index	2.7 ±2.3	2.9 ±1.7	2.8 ±2.0
	(0.9–11.1)	(0.9–7.5)	(0.9–11.1)
SUA, mg/dL	**5.3 ±0.8**	6.6 ±1.0***	6.0 ±1.0
	**(3.7–6.9)**	**(5.5–8.5)**	(4.5–8.5)

BMI, body mass index; FFMI, fat free mass index; FMI, fat mass index; HOMA index, Homeostasis Model Assessment Index; SD, standard deviation; SUA, serum uric acid; * *p* < 0.05, *** *p* < 0.001 vs. women (t-test, Mann-Whitney-U-test).

### Intervention effects on vitamin C and hesperidin metabolites concentrations

Concentrations of vitamin C in plasma and hesperidin metabolites in urine are given in Table 2. Plasma vitamin C levels were increased after one-week consumption of vitamin C-rich beverages, regardless of the presence or absence of hesperidin. This observation did not further increase after the second week, and plasma vitamin C remained significantly elevated. Both the *Hesp*-drink and the *VitC+Hesp*-drink significantly increased the urinary excretion of hesperidin metabolites, irrespective of *VitC*.

**Table 2:** Levels of and changes in levels of plasma vitamin C and of hesperidin metabolites in urine in the ‘HesperidrinC study’

	Time	*Control* (*n* = 20)	*Hesp* (*n* = 20)	*VitC* (*n* = 20)	*VitC + Hesp* (*n* = 20)
		mean ±SE			
Plasma Vit C, µmol/L	T1	35.71 ±1.70	34.94 ±1.71	36.47 ±1.73	35.24 ±1.73
	T2	35.86 ±1.70	**34.16 ±1.71** ^**a**^	41.16 ±1.73	**44.03 ±1.73** ^**a**^
	T3	34.99 ±1.70	**33.68 ±1.71** ^**b**^	41.22 ±1.73	**42.39 ±1.73** ^**b**^
	Δ T2 - T1	0.15 ±1.52	-0.79 ±1.50	4.69 ±1.52*	8.79 ±1.52***
	Δ T3 - T2	-0.87 ±1.50	-0.48 ±1.50	0.05 ±1.52	-1.64 ±1.52
	Δ T3 - T1	-0.72 ±1.60	-1.26 ±1.60	4.75 ±1.62*	7.15 ±1.62***
Total urinary excretion of hesperidin metabolites, µmol/24 h	T1	3.66 ± 1.74	6.18 ± 1.93	5.45 ± 2.42	4.88 ± 2.52
	T3	**2.50 ± 1.67** ^**c**^	**41.60 ± 5.52** ^**c**^	**1.34 ± 1.24** ^**d**^	**56.33 ± 4.99** ^**d**^
	Δ T3 - T1	-1.17 ± 1.95	35.42 ± 5.47***	4.12 ± 2.39	51.45 ± 5.13***

SE, standard error;Linear mixed model corrected for age, gender, Homeostasis Model assessment Index (HOMA)-Index and intervention period: within-group (intervention) changes of outcomes with time are considered significant at *p < 0.05, ***p < 0.001; significant between-group (intervention) differences are indicated by identical superscript characters with ^a^p = < 0.01, ^b^*p* < 0.01, ^c^*p* < 0.001, ^d^*p* < 0.001

**Fig. 3:**
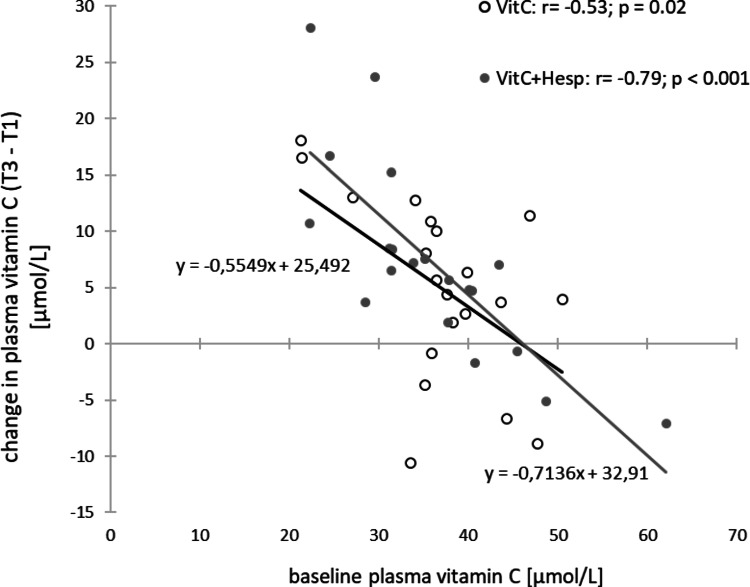
Relationships between change in plasma vitamin C concentration over 2 weeks of intervention and baseline plasma vitamin C concentrations for the *VitC*- and *VitC+Hesp*-intervention (open and grey symbols, respectively)

**Fig. 4:**
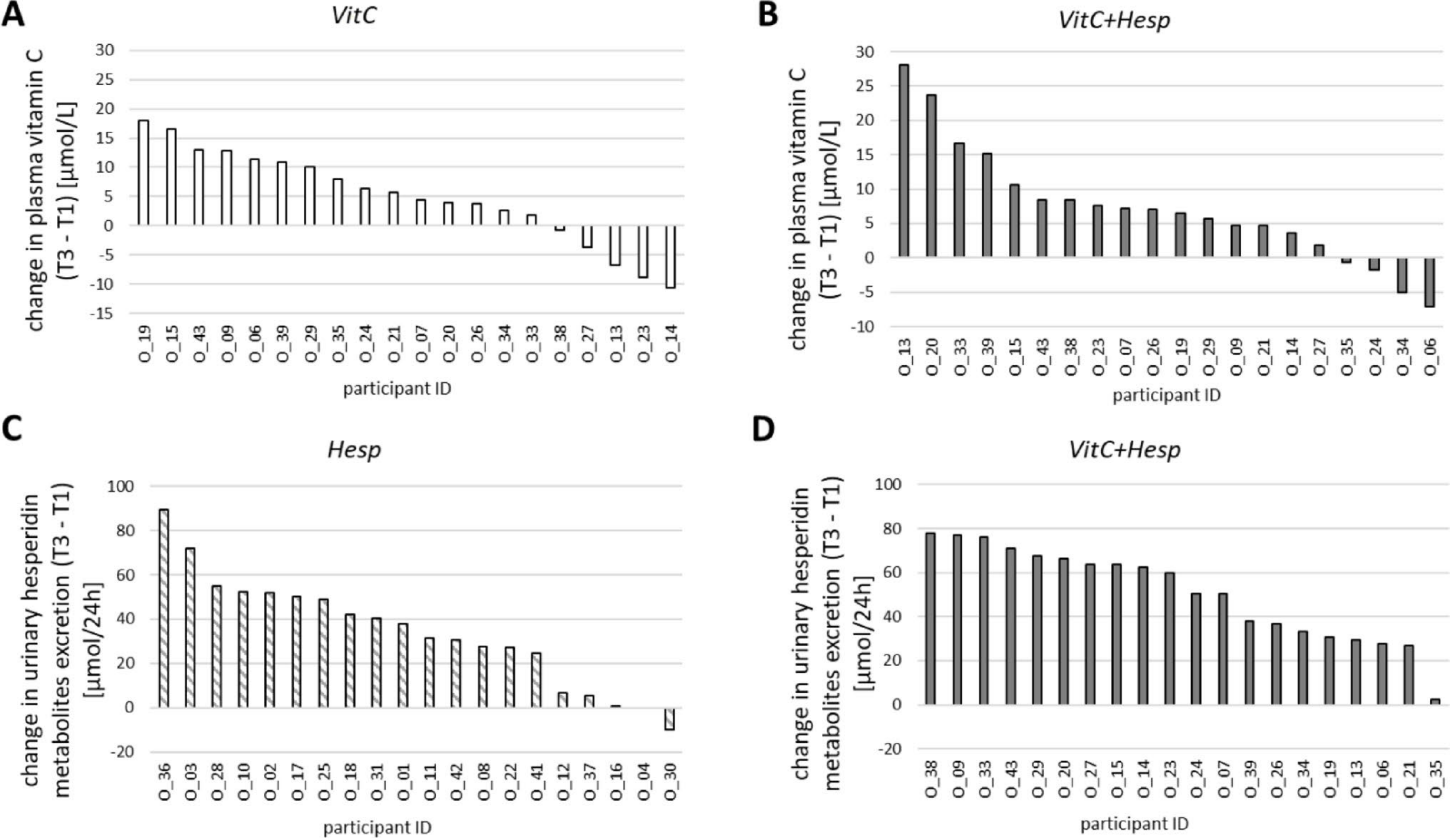
Individual changes in plasma vitamin C over 2 weeks of *VitC-* and *VitC+Hesp*-intervention (Fig. 4A, B) and urinary excretion of hesperidin metabolites over 2 weeks *Hesp-* and *VitC+Hesp*-intervention (Fig. 4C, D)

Lower baseline levels of plasma vitamin C were associated with a greater increase in plasma vitamin C levels after *VitC-* and *VitC+Hesp*-drink (*r* = -0.53; *p* = 0.02 and *r* = -0.79; *p* < 0.001) (Fig. 3). Figure 4 shows individual changes in plasma vitamin C levels (Fig. 4A, B) and urine hesperidin metabolite excretion (Fig. 3C, D) over two weeks of each intervention.

### Intervention effects on uric acid metabolism

Table 3 summarizes SUA levels, UUA, and UAC according to the intervention at different time points. SUA did not differ between groups at any time point. Nonetheless, a significant decrease in SUA was observed during the first week of the intervention with vitamin C-rich drinks (Vitamin C and Vitamin C+Hesp). UUA and UAC did not differ between groups.

**Table 3:** Levels of and changes in uric acid concentration in serum and urine, as well as uric acid clearance in the ‘HesperidrinC study’

	Time	*Control* (*n* = 20)	*Hesp* (*n* = 20)	*VitC* (*n* = 20)	*VitC+Hesp* (*n* = 20)
		mean ±SE			
SUA, mg/dL	T1	5.86 ±0.20	5.85 ±0.20	6.02 ±0.20	5.90 ±0.20
	T2	5.76 ±0.20	5.77 ±0.20	5.54 ±0.20	5.52 ±0.20
	T3	5.86 ±0.20	5.86 ±0.20	5.71 ±0.20	5.69 ±0.20
	Δ T2 - T1	-0.10 ±0.13	-0.08 ±0.13	-0.48 ±0.13**	-0.38 ±0.13*
	Δ T3 - T2	0.10 ±0.13	0.09 ±0.13	0.17 ±0.13	0.17 ±0.13
	Δ T3 - T1	0.0003 ±0.14	0.01 ±0.14	-0.32 ±0.14	-0.21 ±0.14
UUA, g/d	T1	0.51 ±0.03	0.45 ±0.03	0.49 ±0.03	0.46 ±0.04
	T3	0.47 ±0.03	0.45 ±0.03	0.45 ±0.03	0.49 ±0.03
	Δ T3 - T1	-0.03 ±0.03	-0.01 ±0.03	-0.04 ±0.03	0.03 ±0.04
UAC, mL/min	T1	6.13 ±0.39	5.49 ±0.33	5.73 ±0.38	5.32 ±0.46
	T3	5.73 ±0.31	5.47 ±0.40	5.34 ±0.37	5.67 ±0.31
	Δ T3 - T1	-0.40 ±0.30	-0.03 ±0.30	-0.39 ±0.29	0.36 ±0.37

SE, standard error; SUA, serum uric acid; UAC, uric acid clearance; UUA, urine uric acid; linear mixed model corrected for age, gender, Homeostasis Model assessment Index (HOMA) index, and intervention period: within-group (intervention) changes of outcomes with time are considered significant at *p < 0.05, **p < 0.01

**Fig. 5:**
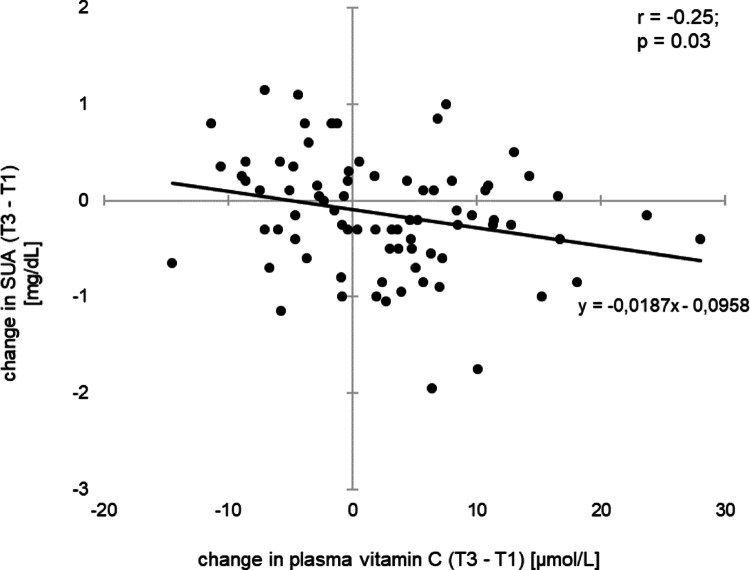
Changes in serum uric acid (SUA) over 2 weeks of intervention by changes in plasma vitamin C levels

There was no association between SUA and plasma vitamin C levels at baseline, whereas SUA was negatively correlated with plasma vitamin C levels after one and two weeks of intervention (*r* = -0.26; *p* = 0.02 and *r* = -0.36; *p* = 0.001). SUA responses to *VitC-* and *VitC+Hesp*-drink varied considerably between individuals (Supplemental Fig. S1). In the total study population, a decrease in SUA correlated negatively with the SUA concentration at baseline (*r* = -0.24; *p* = 0.03). Increase in plasma vitamin C during two weeks of intervention was associated with a decrease in SUA (*r* = -0.25; *p* = 0.03) (Fig. 5). In contrast, there were no correlations between changes in plasma vitamin C levels and changes in UUA and UAC. In addition, there was no association of an increase in excretion of hesperidin metabolites in response to *Hesp*-drinks and changes in SUA, UUA, and UAC.

### Impact of HOMA index on intervention effects

The HOMA index was negatively correlated with plasma vitamin C levels at each of the assessments (T1, *r* = -0.34; *p* = 0.002; T2, *r* = -0.28; *p* = 0.01; T3, *r* = -0.36; *p* = 0.001): The higher the HOMA index, the lower the vitamin C level. However, the HOMA index did not have a significant association with the changes in the plasma levels of vitamin C (data not shown). In addition, the HOMA index did not correlate with urinary hesperidin metabolites or an increase in hesperidin metabolite excretion during the intervention (data not shown).

The HOMA index correlated positively with SUA at each assessment (T1, *r* = 0.31; *p* = 0.005; T2, *r* = 0.36; *p* = 0.001; T3, *r* = 0.35; *p* = 0.002), but did not correlate with changes in SUA, UUA or UAC (data not shown).

## Discussion

The ‘HesperidrinC study’ investigated the uric acid-lowering effect of the orange juice constituents hesperidin and vitamin C, as well as the combination of both. Major findings were: (i) *VitC-*drinks increased plasma vitamin C levels, irrespective of *Hesp* (Table 2; Fig. 4A, B); (ii) SUA decreased following interventions with *VitC*-drinks, irrespective of *Hesp* (Table 3), with larger effects at higher baseline SUA; the decrease in SUA correlated negatively with changes of plasma vitamin C (Fig. 5); (iii) *VitC-*drinks did not affect uric acid excretion; (iv) HOMA index correlated negatively with plasma vitamin C levels and positively with SUA.

### Uric acid

The impact of baseline SUA on the *VitC*-associated decrease in SUA is consistent with the findings from our previous study, which also found more efficient lowering of SUA levels with higher baseline concentrations [11]. Nonetheless, an earlier study had reported that a reduction in SUA in response to a vitamin C intervention was independent of the SUA concentration at baseline [14].

In the present study, a positive correlation was observed between SUA and HOMA index at T1, T2, and T3, while no association was detected between the HOMA index and the change in SUA, UUA, or UAC from T1 to T3. This finding is consistent with the results of earlier studies, which have demonstrated a higher prevalence of high SUA levels with the presence of hyperinsulinemia, IR, and cardio-metabolic risk factors [33–36]. Moreover, experimental data from acute euglycemic hyperinsulinemia conditions indicate that insulin exerts an anti-uricosuric effect [37]. In our study population, people with IR have higher SUA and lower plasma vitamin C levels (see results). It is also conceivable that vitamin C may attenuate the HOMA effect on SUA. Genetic and physiological data from human cell lines have indicated an anti-uricosuric effect of insulin, as it was shown to stimulate reabsorption of uric acid via activation of several urate transporters, especially GLUT9 [38]. While Mendelian randomization analyses have also demonstrated a correlation between elevated SUA and the HOMA index, causality has not been established [39]. The present study’s findings are consistent with the in vitro data from Mandal et al., who had demonstrated the impact of insulin on reabsorptive urate transporters [38].

### Vitamin C and hesperidin metabolites concentrations

Greater increases in plasma vitamin C levels were associated with more marked reductions in SUA levels (Fig. 5). This finding is consistent with the findings of preceding studies, both interventional [14, 40] and observational [16, 41, 42]. The SUA-lowering action of vitamin C has been attributed to the uricosuric effect of vitamin C [13, 14, 41]. However, while *VitC*-drinks increased plasma vitamin C levels and decreased SUA concentrations, there was no uricosuric effect, i.e., UUA or UAC did not increase (Tables 2 and 3). This is in contrast to the previously reported uricosuric effect of vitamin C, which was attributed to the competition between vitamin C and uric acid for a shared renal reabsorption site [43].

The ingestion of 600 mg of vitamin C daily, with or without hesperidin, resulted in a moderate increase in plasma vitamin C concentration after one week, which remained unchanged during the second week of the intervention. The plasma concentration of vitamin C has been demonstrated to exhibit a sigmoidal curve in response to daily oral intake of the vitamin, reaching a plateau at a daily intake of 200 mg [44, 45]. In addition, the bioavailability of vitamin C was complete at 200 mg and decreased at higher intakes. This indicates that the gastrointestinal absorption of vitamin C decreased, while urinary excretion of vitamin C increased consistently with higher intakes [44]. In the present study, daily intake of vitamin C with *VitC*-drinks was approximately 600 mg (i.e., three times the dose described previously). It can be hypothesized that the participants had already reached a plateau after one week and had excreted the excess vitamin C in the urine.

It has been hypothesized that the suggested hypouricemic effect of hesperidin is not, as with vitamin C, the result of increased urinary excretion of uric acid. Rather, this effect is the result of an inhibitory effect on xanthine oxidase and, consequently, reduced uric acid synthesis [17, 18]. Thus far, the available data on the effect of hesperidin on SUA are inconclusive: In a previous study, the effect of similar doses of hesperidin intake (approx. 292 mg per day) to those in the present study was investigated. That study compared the intake of hesperidin from orange juice, which provided an additional 180 mg of vitamin C per serving, with the intake of a supplement containing only hesperidin. The results indicated that orange juice, but not capsuled hesperidin, exhibited a SUA-lowering effect [12]. In another study, it was found that a 12-week daily intake of orange juice (345 mg hesperidin, vitamin C not reported) did not result in a decrease in SUA. However, the consumption of a hesperidin-enriched orange juice (600 mg, vitamin C not reported) led to a substantial reduction in SUA levels [46]. The beverages consumed in the ‘HesperidrinC trial’ differed from those consumed in previous studies in that they contained isolated vitamin C and hesperidin. It has been demonstrated that the solubility of flavanones, in addition to the effects of the food matrix, has a significant impact on the bioavailability of flavanones [47]. Hesperidin is predominantly absorbed in the colon following microbial de-glycosylation and the release of the aglycone hesperetin [48] or metabolized to produce phenyl propionic or benzoic acid derivatives [47]. Post-absorptive metabolism results in the formation of several conjugates, including hesperetin glucuronides and sulfates, which are directly released into the circulation [47].

In the ‘HesperidrinC trial’, an increase in the urinary excretion of hesperidin metabolites was observed following the consumption of the *Hesp*- and the *VitC+Hesp-*drink but not following the consumption of the *Control*- and the *VitC*-drink (Table 2). This finding suggests that the hesperidin from the study drink was bioavailable. The urinary excretion of total hesperidin metabolites was 41.6 ± 5.5 and 56.3 ± 5.0 µmol in 24 h urine after the consumption of the *Hesp-* or *Hesp+VitC-*drink. The relative urinary excretion rates of 10.5% and 14.2% were similar to those reported previously. For instance, Manach et al. (2003) reported the total urinary excretion of hesperidin metabolites, more specifically hesperetin conjugates, to be 15.0 ± 3.5 and 46.7 ± 7.8 µmol after the consumption 0.5 and 1.0 L of orange juice, respectively, with 363 and 730 µmol hesperidin [49]. The present study did not demonstrate a SUA-lowering effect of hesperidin (Table 3). This is in contrast to the results observed in animal models, in which a flavonoid-induced inhibition of xanthine oxidase has been reported [17, 18].

### Strengths and limitations

The major strength of the ‘HesperidrinC trial’ lies in its randomized controlled, double-blind, two-way cross-over, two-week intervention design. In the week preceding, and throughout the two-week intervention study, participants were required to abstain from the consumption of citrus products, vitamin C supplementation, and alcohol, in order to minimize dietary confounding. Accordingly, the study procedure was highly standardized and controlled. As urinary vitamin C excretion has not been assessed in the ‘HesperidrinC trial’, competitive tubular transport at the expense of uric acid excretion could not be ruled out.

Further studies are needed to investigate the role of urinary vitamin C excretion and hesperidin metabolism.

## Conclusion

The study demonstrated that already one week of supplementation with vitamin C was associated with a significant decrease in SUA, whereas there was no effect of hesperidin. There was no uricosuric effect of vitamin C. The present study found that the effects of vitamin C on lowering SUA were more pronounced at higher baseline SUA. The HOMA index correlated positively with SUA but did not correlate with changes in SUA. Given that the average effect of vitamin C on lowering SUA levels is approximately − 0.5 mg/dL, especially in individuals with low baseline levels of vitamin C, it may be advisable to recommend vitamin C supplementation for those who have elevated SUA and low vitamin C levels.

## Supplementary Information

Below is the link to the electronic supplementary material.

Supplementary Material 1

## Abbreviations

BMI: Body mass index
FFMI: Fat free mass index
FMI: Fat mass index
HOMA index: Homeostasis model assessment index
IR: Insulin resistance
SUA: Serum uric acid
UAC: Uric acid clearance
UUA: Urinary uric acid
