# Supplementary material for: Effect of vitamin C and hesperidin on serum uric acid concentrations in healthy adults with high uric acid levels: the randomized controlled ‘HesperidrinC trial’
Source: Eur J Nutr. 2026 Feb 25;65(2):67. doi: 10.1007/s00394-026-03905-z (PMC12935826; doi:10.1007/s00394-026-03905-z)
Supplement: Supplementary file 1 — Supplementary Material 1 [file 394_2026_3905_MOESM1_ESM.docx]

**Effect of vitamin C and hesperidin on serum uric acid concentrations in healthy adults with high uric acid levels - the randomized controlled ‘HesperidrinC trial’**

**European Journal of Nutrition**

Janna Enderle^1^, Rebecca Dörner^1^, Daria Tondar^1^, Mario Hasler^2^, Caroline Gilcher^3^, Christof B Steingass^3^, Ralf Schweiggert^3^, Manfred J Müller^1^, Anja Bosy-Westphal^1^*

^1^Institute of Human Nutrition and Food Science, Christian-Albrechts University Kiel, Kiel, Germany

^2^Applied Statistic, Agricultural and Food Economics Faculty, Christian-Albrechts University Kiel, Kiel, Germany

^3^Chair of Analysis and Technology of Plant-based Foods, Department of Beverage Research, Geisenheim University, Geisenheim, Germany

* Corresponding author: Prof. Dr. Dr. A. Bosy-Westphal, Institut für Humanernährung, Christian-Albrechts-Universität Kiel, Düsternbrooker Weg 17, D 24105 Kiel, abosyw@nutrition.uni-kiel.de


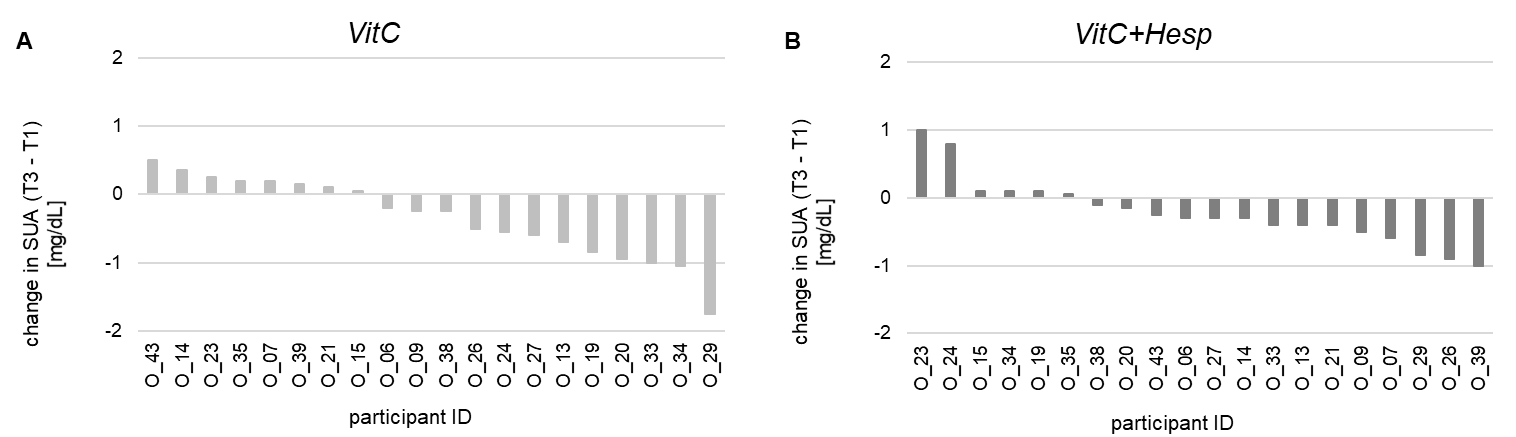
**Fig. 1S** Individual changes of serum uric acid (SUA) over 2 weeks of VitC- (**Fig. 1SA**) and *VitC+Hesp*-intervention (**Fig. 1SB**)

**Table S1** Ingredients of the four different study drinks of the ’HesperidrinC study’

| Ingredient per 200 mL study drink | *Control* | *Hesp* | *VitC* | *VitC + Hesp* |
| --- | --- | --- | --- | --- |
| L-ascorbic acid | - | - | 592 ±12 mg | 591 ±13 mg |
| Hesperidin | - | 242 ±6 mg (397 ±10 µmol) | - | 241 ±7 mg (396 ±12 µmol) |
| Glucose | 4.4 g | 4.4 g | 4.4 g | 4.4 g |
| Fructose | 4.4 g | 4.4 g | 4.4 g | 4.4 g |
| Sucrose | 9.6 g | 9.6 g | 9.6 g | 9.6 g |
| Citric acid | 1.4 g | 1.4 g | 1.4 g | 1.4 g |
| Safflower extract | 80.1 mg | 80.1 mg | 80.1 mg | 80.1 mg |
| Sunset yellow | 0.4 mg | 0.4 mg | 0.4 mg | 0.4 mg |
| Clouding agent | 0.92 g | 0.12 g | 0.92 g | 0.12 g |
| Natural orange aroma | 0.03 mL | 0.03 mL | 0.03 mL | 0.03 mL |

**Figure S2** CONSORT Flow-Chart of included and excluded participants over the course of the study.

**CONSORT 2010 Flow Diagram**

## Follow-Up

Analysed (n=20)
♦ Excluded from analysis (give reasons) (n=1^³^)

## Analysis

Analysed (n=20)
♦ Excluded from analysis (give reasons) (n=0)

Lost to follow-up (give reasons) (n=0)

Discontinued intervention (give reasons) (n=2^²^)

Lost to follow-up (give reasons) (n=0)

Discontinued intervention (give reasons) (n=0)

## Enrollment

Allocated to intervention (n=21)

♦ Received allocated intervention (n=21)

♦ Did not receive allocated intervention (give reasons) (n=0)

## Allocation

Allocated to intervention (n=22)

♦ Received allocated intervention (n=21)

♦ Did not receive allocated intervention (give reasons) (n=1^1^)

Randomized (n=43)

Excluded (n=64)

♦  Not meeting inclusion criteria (n=63)

♦  Declined to participate (n=1)

♦  Other reasons (n=0)

Assessed for eligibility (n=107)

**D**

^1^ One participant declined participation before the first treatment.

^2^ One participant developed an irritant skin reaction.

³ Incidental finding: Diabetes Type 2 – not meeting inclusion criteria
